# Understandings and critiques of biocultural diversity conservation and future recommendations for conservation actors

**DOI:** 10.1111/cobi.70131

**Published:** 2025-08-24

**Authors:** Natalie D. L. York

**Affiliations:** ^1^ Global Development Institute The University of Manchester Manchester UK

**Keywords:** biocultural diversity, conservation, culture, Indigenous Peoples and local communities, political ecology, reflexivity, conservación, cultura, diversidad biocultural, ecología política, pueblos indígenas y comunidades locales, reflexión, 生物文化多样性, 保护, 土著人民和当地社区, 文化, 反思性, 政治生态学

## Abstract

As biocultural approaches to conservation gain traction (e.g., through international commitments to Indigenous Peoples and local communities) and external conservation actors increasingly seek to engage with on‐the‐ground holders of biocultural diversity, improved understanding is needed of what biocultural diversity means. Building on the foundation provided by Bridgewater and Rotherham, I appraised how biocultural diversity conservation has been framed and critiqued in the academic literature based on a thematic analysis of 95 papers. Biocultural diversity was understood through the concepts of biocultural diversity hotspots; Indigenous and local knowledge; cultural landscapes; the roles and rights of Indigenous Peoples and local communities; biocultural identity; and urban people–nature interactions. Four criticisms of the concept were identified, including a focus on conserving tradition, risk of overgeneralization, neglect of biocultural conflicts, and a lack of attention to power dynamics. A political ecology perspective on biocultural diversity could help address these criticisms by encouraging external conservation actors to reflect on specific questions about power when engaging with holders of biocultural diversity. If external conservation actors are willing to engage in this reflexive practice, for example, by following the prompts I devised (e.g., What preconceptions do external actors hold about culture and identity in this context? Are external actors expecting people to live or behave traditionally? Do Indigenous Peoples and local communities have decision‐making power?), commitments to conserving biocultural diversity could achieve global biodiversity goals while upholding social and environmental justice principles.

## Abstract (es)

Comprensión y crítica de la conservación de la diversidad biocultural y recomendaciones para los actores de la conservación

**Resumen:**

A medida que los enfoques bioculturales de la conservación ganan terreno (p. ej., a través de compromisos internacionales con los pueblos indígenas y las comunidades locales) y los actores externos de la conservación buscan cada vez más colaborar con los poseedores de la diversidad biocultural, es necesario comprender mejor qué significa la diversidad biocultural. A partir de la base proporcionada por Bridgewater y Rotherham (2019), evalué cómo se ha enmarcado y criticado la conservación de la diversidad biocultural en la literatura académica con base en un análisis temático de 95 artículos. La diversidad biocultural se define con los conceptos de puntos calientes de diversidad biocultural, conocimientos indígenas y locales, paisajes culturales, las funciones y los derechos de los pueblos indígenas y las comunidades locales, identidad biocultural e interacciones entre las personas y la naturaleza en las zonas urbanas. Identifiqué cuatro críticas al concepto, entre ellas el énfasis en la conservación de la tradición, el riesgo de generalización excesiva, el descuido de los conflictos bioculturales y la falta de atención a las dinámicas de poder. Una perspectiva de ecología política sobre la diversidad biocultural podría ayudar a abordar estas críticas al animar a los actores externos de la conservación a reflexionar sobre cuestiones específicas relacionadas con el poder cuando interactúan con los poseedores de la diversidad biocultural. Si los actores externos de la conservación están dispuestos a participar en esta práctica reflexiva mediante las indicaciones que he elaborado (p. ej., ¿Qué ideas preconcebidas tienen los actores externos sobre la cultura y la identidad en este contexto? ¿Los actores externos esperan que las personas vivan o se comporten de forma tradicional? ¿Los pueblos indígenas y las comunidades locales tienen poder de decisión?), los compromisos con la conservación de la diversidad biocultural podrían alcanzar los objetivos mundiales de biodiversidad al tiempo que se defienden los principios de justicia social y ambiental.

## 对生物文化多样性保护的理解与批判及对保护行动者的未来建议

**【摘要】** 随着生物文化保护方法越来越得到认可(如通过国际社会对土著人民和当地社区的承诺), 外部保护行动者越来越希望与实际拥有生物文化多样性的社区开展合作, 因此也需要更好地理解生物文化多样性的内涵。基于 Bridgewater 和 Rotherham (2019) 的研究基础, 本文通过对 95 篇论文进行主题分析, 评估了学术文献中对生物文化多样性保护的框架构建与批判。人们一般通过以下概念来理解生物文化多样性:生物文化多样性热点地区;土著人民和当地知识;文化景观;土著人民和当地社区的角色与权利;生物文化身份;以及城市中的人与自然互动。本研究确定了对该概念的四条批判, 包括过分强调对传统的保护、存在过度泛化的风险、对生物文化冲突的忽视, 以及缺乏对权力动态关注。在生物文化多样性中纳入政治生态学的视角, 有利于鼓励外部保护行为者在与拥有生物文化多样性的社区进行互动时反思与权力相关的问题, 以应对这些批评。如果外部保护行为者愿意参与这种反思性实践(例如遵循研究者设计的提示:外部行为者在此情境下对文化与身份有什么先入之见？外部行为者是否期望人们以传统方式生活或行动？土著人民和当地社区是否拥有决策权？), 则对生物文化多样性的保护承诺既有助于实现全球生物多样性目标, 又能维护社会和环境正义原则。 **【翻译: 胡怡思; 审校: 聂永刚】**

## INTRODUCTION

Trends in global conservation agendas and priorities reflect an ongoing shift in how people's relationships with nature are understood (Friedman et al., 2022). New policies and commitments are changing the ways that the roles of Indigenous Peoples and local communities are recognized and valued by global conservation actors. The Global Biodiversity Framework (GBF) brings together biodiversity loss, ecosystem restoration, and protection of Indigenous Peoples’ rights as key interrelated priorities, and the Glasgow Climate Pact pledged US$1.7 billion to conservation activities led by Indigenous Peoples and local communities (UK National Archives, 2023). Global‐scale investments, such as the Global Environment Facility's (GEF) Inclusive Conservation Initiative, provide direct financing to several Indigenous and local community organizations to support and strengthen their stewardship over biodiverse landscapes and seascapes (ICI, 2024).

Alongside international commitments, there has been a revival of research on conserving biocultural diversity. Biocultural diversity is a boundary concept that captures the links and feedbacks between human cultural diversity and biological diversity based on diverse Indigenous epistemologies and extensive advocacy efforts (Bridgewater & Rotherham, 2019; Lukawiecki et al., 2022). It is created and conserved in situ by on‐the‐ground actors, including many Indigenous Peoples and local communities around the world, as they interact with and shape nature through sociocultural practices (Agnoletti & Rotherham, 2015; Bridgewater & Rotherham, 2019; Cocks, 2006). These actors have long pursued means of protecting and restoring their biocultural diversity, for example, by establishing social movements, seeking compensation for damage from industrial actors, and securing greater recognition of their rights, including through struggles for territorial sovereignty and self‐determination (e.g., Aini et al., 2023; Youdelis et al., 2021).

Recent research suggests that a biocultural diversity framework could help achieve the Convention on Biological Diversity's (CBD) 2050 vision of living in harmony with nature (Reyes‐García et al., 2023). Under the current framing of biodiversity conservation, there is a risk that the GBF Target 3, which aims to conserve 30% of land, waters, and seas by 2030 (CBD, 2022), will encourage further adoption of exclusionary and militarized conservation techniques, as the global protected area network is expanded (Kashwan et al., 2021). In contrast, biocultural approaches to conservation focus on achieving “effective and just conservation outcomes while addressing erosion of both cultural and biological diversity” (Gavin et al., 2015, p. 140). Shifting focus to biocultural conservation could move this vision beyond the human–nature dichotomy and place value on the ways many Indigenous Peoples and local communities contribute to conservation outside formal protected areas.

There has been considerable debate around the meaning of *biocultural diversity* and the concept's applicability in conservation settings (e.g., Diaz‐Reviriego et al., 2024; Franco, 2022; Hughes & Vadrot, 2019). In their seminal piece on biocultural diversity's role in nature and heritage conservation, Bridgewater and Rotherham (2019) present a comprehensive overview of how biocultural diversity has evolved and gained traction across academic research, policy, and practice. Biocultural diversity initially attracted international scientific attention following the Declaration of Belém (1988), which recognizes “there is an inextricable link between cultural and biological diversity.” The term itself was used to explain these links and incorporate discussions of nature and cultural heritage in conservation policy, for example, through notions of cultural keystone species (Garibaldi & Turner, 2004) and cultural landscapes (Nassauer, 1995), and to develop tools, such as biocultural community protocols (Bridgewater & Rotherham, 2019). The concept is recognized at global policy scales, including in the Intergovernmental Science–Policy Platform on Biodiversity and Ecosystem Services (IPBES) conceptual framework (Diaz et al., 2015).

As attention to biocultural diversity grows and global‐level funding becomes available for biocultural conservation (e.g., GEF‐7 Inclusive Conservation Initiative), external actors are increasingly engaging with on‐the‐ground holders of biocultural diversity. Although the agency of local actors is key to the success or failure of any intervention, there are risks that attempts by external actors to influence biocultural conservation may undermine local work. Political ecologists have been calling for better attention to the power and politics of environmental change for decades (Adams & Hutton, 2007; Bryant, 1991; Jones, 2006). This rich body of work demonstrates how by understanding that “ideas about nature…are formed, shared and applied in ways that are inherently political” one can begin to critically question conservation policies, their assumptions, and their implications (Escobar [1998] in Adams & Hutton [2007, p. 149]). For instance, strict protected areas have received extensive criticism for displacing communities in favor of pristine nature, threatening rights and livelihoods, and fueling social conflicts (Adams & Hutton, 2007; Neumann, 1992), whereas community conservation initiatives such as the Communal Areas Management Programme for Indigenous Resources (CAMPFIRE) in Zimbabwe have been criticized for failing to genuinely place local livelihoods at the core of conservation efforts (Jones, 2006). To understand the potential risks associated with external actors influencing biocultural diversity conservation, greater clarity on how the concept of biocultural diversity is used and critiqued in conservation circles is needed.

I built on Bridgewater and Rotherham's (2019) work by conducting a critical appraisal of the ways biocultural diversity is framed and critiqued in the academic literature, based on a thematic analysis of 95 papers on biocultural diversity conservation. I explored key understandings and critiques of biocultural diversity and devised a series of guiding prompts to address these criticisms.

## METHODS

I reviewed the academic literature on biocultural diversity and environmental conservation. I searched the databases Scopus and Web of Science on 25 August 2022 for papers published from 1990 to 2023 in peer‐reviewed journals and written in English. I used the following search terms: “*biocultural diversity*” combined using the Boolean operator AND with contextual terms separated by the Boolean operator OR: *conservation*, *restoration*, *environment*, and *biodiversity*.

The first search returned 216 papers. A second search on 27 November 2023 identified 82 additional papers, and 8 papers were identified from searches of the reference lists of key papers. In total, 306 papers were screened for inclusion.

I screened papers for inclusion in a 2‐stage process based on titles and abstracts and then examined the full texts. The general inclusion criteria were articles published in a peer‐reviewed journal, written in the English language, and linked to environmental conservation. Specifically, articles had to have a central focus on biocultural diversity or present a conceptual framework for biocultural diversity; define biocultural diversity when used in the text; or suggest in the abstract that the full‐text paper did do so.

Interrater agreement was calculated on a 10% subset of the papers with an independent reviewer at the title and abstract screening stage with the Cohen's kappa calculation. I amended the inclusion criteria until Cohen's kappa reached an acceptable level of agreement (>0.6) between researcher and independent reviewer (0.64). This acknowledges that a degree of subjectivity is unavoidable when reviewing qualitative data (Mallett et al., 2012). An audit trail was kept in Excel to ensure trustworthiness for qualitative validity assessment (Anfara et al., 2002; Lincoln & Guba, 1985).

**FIGURE 1:**
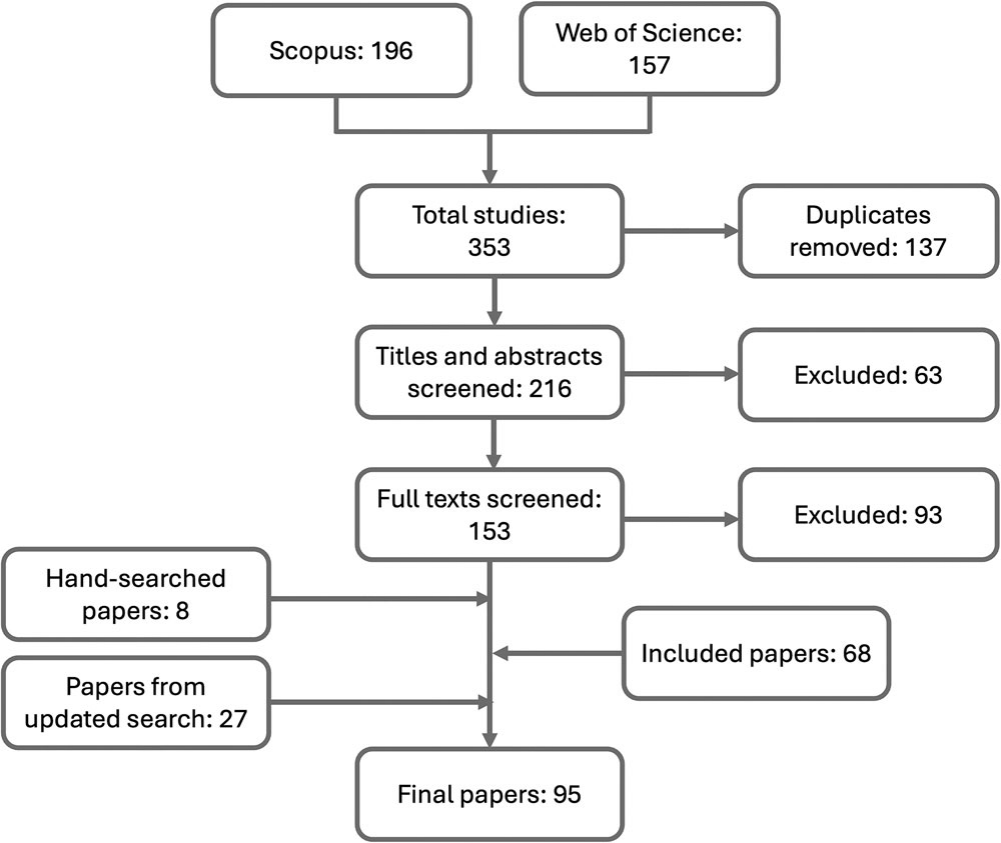
Outline of the full screening process undertaken for a review of academic literature on biocultural diversity and environmental conservation.

Following screening, 95 papers remained for thematic analyses (Figure 1; S1). Based on methodological guidance for qualitative reviews by Cherry et al. (2017), I extracted data from each paper with a predeveloped framework (Table 1). This framework was designed to extract sections of each paper containing understandings of biocultural diversity. I recorded contextual information for each paper and specific applications or critiques of the concept. I then coded the data inductively following Coffey and Atkinson's (1996) open coding approach to draw connections across different authors’ understandings of biocultural diversity, initially recording primarily in vivo codes sorted into similar sets to generate themes regarding key understandings or critiques (Table 2).

**TABLE 1:** Data extraction framework used to extract data from each paper for the thematic analyses of papers on biocultural diversity and environmental conservation.

Code	Description
Key objective	What is the primary objective of the paper?
Biocultural diversity	How does the paper define *biocultural diversity*? Does it offer a framework for biocultural diversity?
Core, peripheral, or negligible	How significant is biocultural diversity to the focus of the paper?
Conservation	Which element of conservation does the paper focus on? How does it relate biocultural diversity to conservation?
Core, peripheral, or negligible	How significant is conservation to the focus or context of the paper?
Geographic scope	Is the research focused on a specific geographic location? If so, where?
Additional comments	What additional information is relevant (e.g., critiques, applications of biocultural diversity)?

**TABLE 2:** Inductive codes and overarching themes generated through thematic analysis.

Themes	Codes
Key understandings	
Biocultural diversity hotspots	Biological, cultural, and linguistic diversity Cultural keystone species Coevolution Common threats Correlations Diversity of life Inextricable links Place based Feedbacks Geographic overlap Hotspots Island–coastal regions Index Mapping Measure Language richness Language vitality Linguistic extinction Spatial Socioecological adaptive systems Tropical regions
Indigenous and local knowledge	Botanical knowledge Ethnobiology Ethnospecies Farmers’ wisdom Food‐based knowledge Indigenous knowledge Interdependences Language encodes knowledge Linguistic diversity Local ecological knowledge Medicinal plants Safeguarding knowledge Seeds Traditional ecological knowledge Traditional food practices Wild plants
Roles and rights of Indigenous Peoples and local communities	Advocacy Biocultural community protocols Biocultural memory Coexistence Conflict with conservation Cultural practices Conservation or development objectives Cosmologies Diverse values Endogenous development Endogenous processes Holistic worldviews Indigenous Peoples Indigenous rights Interdependencies Institutions Kinship Local communities Mutually supportive ICCAs Interrelationships Governance Human rights
	Pluralistic worldviews Protective mechanisms Reciprocity Resource management Responsibilities Rules Sacred sites Sacred species Self‐determination Social justice Spiritual, religious values Stewardship Subsistence Territorial rights Traditions Well‐being Worldviews
Cultural landscapes	Agrobiodiversity Agroecosystems Coevolution Cultural heritage Cultural practices Cultural landscapes Eco‐cultural landscapes Food systems Historical practices Hybrid nature–culture systems Human influence on landscape Mosaic Satoyama Spatial diversity Species to ecosystems Rural landscapes Traditional agricultural landscapes Traditional management practices
Biocultural identity	Collective memory Creativity Cultural traditions Emotions Heritage Identity Intangible Music Place attachment Relationships with species Sense of place Social identity
Urban people–nature interactions	Beyond pristine Cities Conscious engagement Cultural ecosystem services Diverse value systems Ecosystem services Global North Lived experiences Living with biodiversity Multicultural Novel ecosystems Peri‐urban Place making Reciprocity Stewardship
	Sustainability Urban biocultural diversity Urban forests Urban green space Urban markets Urban planning Urban plant use Use value of resources Well‐being
Criticisms	
Focus on conserving tradition	Adaptive Allowing cultural systems to adapt Culture as dynamic, evolving, flexible External expectations Globalizing Indigenous urban knowledge Modernization Moving beyond tradition Narrow notions of identity Tradition as problematic, colonial
Overgeneralization and loss of advocacy function	Appropriating biocultural diversity concept Diluting the concept Imperializing conceptual territory
Neglect of biocultural conflicts	Biocultural conflicts Harmful cultural practices
Lack of attention to power	Controlling Indigenous interests Emancipatory potential Hierarchy Institutions Power relations perspective is needed Reinforcing colonizing scholarship Reinforcing marginalization of rural local communities Vulnerability of cultural groups

Although comprehensive, this review offers a representation of how biocultural diversity is understood within the English language, peer‐reviewed literature, and not beyond this. It is important to acknowledge this limitation because biocultural diversity is being theorized across different languages (e.g., Díaz‐Reviriego et al., 2024), but this is not reflected in this review. In addition, although it is common for reviews to restrict their inclusion criteria to peer‐reviewed papers, this quality assessment strategy meant that reports, websites, and several key books on biocultural diversity were excluded (e.g., Maffi, 2001; Maffi & Woodley, 2012; Rozzi et al., 2018), as were biocultural initiatives not represented in the academic literature (e.g., Terralingua or Local Biodiversity Outlooks).

Although the academic literature on biocultural diversity conservation contains significant contributions from Indigenous Peoples and local communities, including as advocates, researchers, and research participants (e.g., Aini et al., 2023; Dyrset et al., 2022; Gutiérrez‐Santillán et al., 2019; Nemogá et al., 2022; Sirakova, 2023), a review of academic literature alone cannot provide sufficient insight into the perspectives of on‐the‐ground holders of biocultural diversity. Instead, as external actors increasingly seek to engage with biocultural conservation, I sought to provide insight into how biocultural diversity is understood and critiqued within the academic literature, providing recommendations for external actors on how to mitigate the reproduction of problematic dynamics. However, my review is limited in its relevance to these external actors and does not provide recommendations for on‐the‐ground practitioners of biocultural conservation themselves. Lukawiecki et al. (2022, p. 375) stress that systematic reviews risk “presenting colonised worldviews if left unexamined” by excluding knowledge systems that sit outside academic literature. This critique also applies to reviews that consider papers in only one language—particularly the English language due to its colonial legacies (Fortier, 2018)—as I have noted. My findings should be interpreted with these points in mind.

## RESULTS

Understandings of biocultural diversity conservation were used initially to represent geographical areas with high levels of linguistic and biological diversity and evolved into a multifaceted concept with relevance for conservation (Bridgewater & Rotherham, 2019). The thematic analysis revealed 6 key ways in which biocultural diversity is understood in the academic literature on conservation: biocultural diversity hotspots; Indigenous and local knowledge (ILK); cultural landscapes; the roles and rights of Indigenous Peoples and local communities; biocultural identity; and urban people–nature interactions. Although each theme reflected a distinct understanding of biocultural diversity, connections existed between these themes, which is the nature of qualitative data (e.g., Braun & Clarke, 2006).

### Biocultural diversity hotspots

From the late 1990s onward, as biocultural diversity research gained ground, scholars focused on identifying the correlations between biological and linguistic diversity (Maffi, 2005), based on observations that language extinctions were happening in parallel with biodiversity losses (Maffi, 2002). Although biological diversity and linguistic diversity were initially discussed as separate entities, anthropologists Luisa Maffi (2005) and David Harmon (2007) popularized use of the combined term biocultural diversity, taking linguistic richness as a proxy for cultural diversity. Based on these correlations and geographical overlaps, Harmon (2007) suggested extending the concept of biodiversity hotspots to biocultural diversity hotspots. Efforts to map the correlations between biodiversity and linguistic diversity hotspots are still ongoing (Vidal & Brusca, 2020), mostly identified in tropical regions (Briggs et al., 2019) and sometimes including island or coastal regions (Hong et al., 2018), although Pert et al. (2015) noted that causation is not well understood. As well as linguistic diversity, language vitality is now considered key to biocultural diversity (Reyes‐Garcia et al., 2023).

One of the most cited definitions of *biocultural diversity* was the “diversity of life in all its manifestations—biological, cultural, and linguistic—which are interrelated within a complex socioecological adaptive system” (Maffi, 2005, p. 602). Although the terms *biocultural* and *biolinguistic diversity* were used interchangeably at first, biocultural diversity is thought to reflect understandings of cultural diversity as more complex than the number of languages spoken in a place (Maffi, 2005). Meanings of cultural diversity have since been extended to include economic diversity, lifestyle diversity (including diets and land management practices), artistic diversity (e.g., specific architecture, traditional crafts, and music) (Tydecks et al., 2023), and seasonal life cycles that can be organized in biocultural calendars (Rozzi et al., 2023). Cultural keystone species were also linked to biocultural diversity, as markers of cultural diversity (Axelsson & Franco, 2023; Chengere et al., 2022; Garibaldi & Turner, 2004; Min et al., 2022; Reyes‐García et al., 2023).

Early definitions of *biocultural diversity* were often cited and built on in academic literature (Apgar et al., 2011; Boillat et al., 2013; Davidson‐Hunt et al., 2012; Dobrovodská et al., 2019; Hill et al., 2011; Hong et al., 2018; Latorre et al., 2018; Nemogá et al., 2022; Pert et al., 2015) and were used to inform frameworks by IPBES (Díaz et al., 2015) and the United Nations Educational, Scientific and Cultural Organisation (UNESCO) (Persic & Martin, 2008). Understandings of the concept moved beyond identifying geographical overlaps between biological and linguistic diversity to explore the dynamic links and feedbacks between cultural and biological diversity (Bridgewater & Rotherham, 2019; Liu et al., 2023). Rozzi (2012, p. 50) presented biocultural ethics as a normative extension of biocultural diversity “that demands the recovery of forgotten or negated biophysical and biocultural realities.” The term *biocultural homogenization* was introduced to capture biocultural diversity loss, which was largely attributed to rural–urban migration patterns leading to cultural severance (Romero Puentes & Rodriguez Susa, 2022; Rotherham, 2013; Rozzi, 2012; Spirito et al., 2022).

### Indigenous and local knowledge

Biocultural diversity studies have been a core focus in the field of ethnobiology since the First International Congress of Ethnobiology in 1988. This congress led to the Declaration of Belém, which is a commitment made “to stop the ongoing and often dramatic decline in global diversity of both nature and culture” (Bridgewater & Rotherham, 2019, p. 292). Lukawiecki et al. (2022) noted that although the term *biocultural diversity* itself was not used in the declaration, the concept is widely considered to have originated there. I found that although many ethnobiology studies focus on investigating biocultural diversity, there is often limited conceptualization within these studies about the concept's meaning (e.g., Moreno‐Calles et al., 2016).

Nonetheless, from an ethnobiology perspective, communities’ botanical knowledge is often understood to be an indicator of biocultural diversity (Latorre et al., 2018). In the academic literature, a variety of terms were used to describe these diverse forms of knowledge, but ILK, traditional ecological knowledge (TEK), and local ecological knowledge (LEK) were most common. In this context, knowledge was understood as a system, “drawn from lived experiences of people throughout their history of interaction with the environment…place‐specific, non‐scientific, informal knowledge that is passed down the generations through cultural practices and traditions” (Dyrset et al., 2022, p. 133).

Here, linguistic diversity was considered a marker of biocultural diversity due to the role of languages in containing ILK (Cocks & Wiersum, 2014; Singh et al., 2010), which can incorporate many levels of cultural knowledge and links with nature and is often associated with agrobiodiversity (Bridgewater & Rotherham, 2019). Agrobiodiversity is a subset of biodiversity that encompasses “the variety and variability of animals, plants and micro‐organisms that are used directly or indirectly for food and agriculture, including crops, livestock, forestry, and fisheries” (FAO, 2004, p. 2). Knowledge about wild plants as food, for instance, can be key for building community‐specific responses to environmental and social transitions (Prakofjewa et al., 2023). In this way, biocultural diversity conservation is key for food system resilience and security (Argumedo et al., 2021). Traditional food practices, such as yoghurt cultivation, are also thought to maintain biocultural diversity at the microbial level, and loss of these practices that preserve microbial diversity has been linked to the increasing emergence of epidemics (Sirakova, 2023).

Studies that focus on ILK tended to highlight biocultural diversity at risk. Kulak et al. (2022) explained how language loss is linked to the loss of unique medicinal plants, uses, and meanings, particularly among communities who practice oral and land‐based teachings. As language encodes knowledge, for example, through folk taxonomies (Soldal et al., 2023), some suggested that language loss is likely to be even more critical to the extinction of medicinal knowledge than biodiversity loss (Cámara‐Leret & Bascompte, 2021). In contrast, oral knowledge transmission can be an important indicator that biocultural diversity is prevailing, such as the continuation of knowledge about stingless beekeeping practices in Mexico (Simms & Porter‐Bolland, 2022). In light of this indicator, there were calls to conserve diverse forms of ILK in order to conserve biocultural diversity (Singh et al., 2010). Agrawal (2005) highlighted how ethnobiology has long had an apolitical focus on documenting threatened ILK, without addressing the underlying drivers that threaten the ways of lives of diverse Indigenous Peoples and local communities. Marrero et al. (2023, p. 2) argued that, in the case of small island food systems, a critical approach is needed to truly conserve biocultural diversity, “one that asks not only what traditional ecological knowledge is but also who holds it, with what power, and with what desire to continue holding it amid existential threat.”

### Roles and rights of Indigenous Peoples and local communities

Conventional understandings of biocultural diversity had “a strong, almost exclusive, focus on Indigenous Peoples and local communities” (Davidson‐Hunt et al., 2012, p. 4). At the surface level, this reflected geographic associations between the diversity of Indigenous Peoples’ languages and plant diversity in tropical regions; high levels of biocultural diversity are often found in Indigenous Peoples’ territories (Apgar et al., 2011). As a result, biocultural diversity was understood as primarily represented by Indigenous Peoples around the world (Briggs et al., 2019; Linares‐Rosas et al., 2021). Beyond this, there were also strong philosophical links between biocultural diversity and the diverse worldviews of Indigenous Peoples. This included emphasis on relational values (Monroy‐Sais et al., 2022) reflected in coevolutionary relationships between communities and their land (Apgar et al., 2011) and understandings of culture and nature as intrinsically interlinked (Oloriz & Parlee, 2020; Pert et al., 2015; Singh et al., 2010; Vidal & Brusca, 2020). Biocultural diversity is considered part of the memory and distinct biocultural heritage of many Indigenous Peoples (Gutiérrez‐Santillán et al., 2019).

In the biocultural diversity literature, Indigenous Peoples and local communities were often recognized as playing an active role in managing and safeguarding the world's biocultural diversity (Bridgewater & Rotherham, 2019; Hill et al., 2019; Linares‐Rosas et al., 2021). For example, Cocks et al. (2018) described how places regarded as sacred by Indigenous Peoples around the world are often protected as sanctified or ceremonial locations, where sacred species are protected in tandem. Based on this, biocultural diversity was often thought of in terms of its existence in and protection under Indigenous Peoples and local communities and threats to it from environmental degradation and exploitation (Bridgewater & Rotherham, 2019; Cocks et al., 2018). Turner et al. (2022) suggested that biocultural diversity can also be a useful tool for helping to amplify diverse Indigenous practices and perspectives, such as Indigenous systems of governance and knowledges as models for climate change adaptation, although there is also a long‐held tradition that applies the concept more broadly (e.g., Cocks, 2006).

These assumptions about Indigenous Peoples and local communities as environmental guardians are not unproblematic because fortress‐style conservation interventions in many areas of the world have led to Indigenous Peoples’ exclusion from their ancestral lands (Brockington & Igoe, 2006). Many core areas of biocultural diversity were characterized by contested territorial rights and threatened by land dispossession and largescale development (Apgar et al., 2011). For instance, Buergin's (2015) work with Karen communities in northern Thailand highlights how biocultural diversity can manifest as conflict between local livelihood and identity claims versus national or global conservation interests. This suggests that the biocultural diversity literature may have romanticized the links between Indigenous Peoples and biocultural diversity conservation to an extent, overlooking associated struggles or tensions. Biocultural diversity also received criticism for its focus on protecting traditional ways of living as a marker of cultural diversity, and there were calls for the concept to move beyond this and recognize that many Indigenous Peoples participate in the globalizing world (Arts et al., 2018; Mooij et al., 2019). These criticisms reflected a reminder by Davidson‐Hunt et al. (2012) that biocultural diversity itself is not an Indigenous concept; rather, it was developed by scholars to understand the links between nature and culture. Biocultural diversity also has been criticized for emphasizing Indigenous Peoples over local communities, which has encouraged extension of the concept to include diverse peasant communities (Aldasoro et al., 2023).

Keeping these critiques in mind, biocultural diversity nonetheless had strong conceptual links with Indigenous Peoples’ rights and advocacy work (Lukawiecki et al., 2022). Nemogá et al. (2022, p. 1) advocate framing biocultural diversity as “an essential tool for the development of culturally appropriate protective mechanisms for ILK.” For example, in cases reported from Colombia and the Australian humid tropical forests, biocultural diversity provided an effective advocacy tool for protecting Indigenous Peoples’ rights and knowledges through the development of biocultural community protocols (Hill et al., 2011; Nemogá et al., 2022). Moreover, biocultural diversity conservation was sometimes explicitly linked to self‐determination and assertions of territorial rights (Davidson‐Hunt et al., 2012). Apgar et al. (2011, p. 557) argued that endogenous processes—“spiritual and cultural worldviews and livelihood practices…rooted in Indigenous understanding of the cosmos”—are more critical for biocultural diversity conservation than statutory biocultural designations, such as Indigenous and community conserved areas (ICCAs), because the latter do not always fully recognize local governance (Apgar et al., 2011).

Davidson‐Hunt et al. (2012) emphasized the importance of Indigenous Peoples’ and local communities’ control over their lands and resources in order for endogenous processes, and in turn biocultural diversity, to be protected. Marrero et al. (2023) positioned stewardship as the active process through which biocultural diversity can be conserved. This was also highlighted in Gavin et al.’s (2015) guidelines for biocultural approaches to conservation that state the need to recognize rights to autonomy and self‐determination for Indigenous Peoples and local communities. For biocultural conservation to be effective, local perspectives must be recognized as favoring biodiversity according to their own understanding of what diversity is (Franco‐Moraes et al., 2023). Gavin et al. (2018) stressed a need to recognize multiple worldviews and knowledge systems, calling for dynamic, pluralistic, and partner‐based approaches to conservation.

### Cultural landscapes

Cultural or ecocultural landscapes were also a key concept for understanding biocultural diversity, providing a lens for understanding how “culture changes landscapes and culture is embodied by landscapes” (Nassauer, 1995, p. 229; Bridgewater & Rotherham, 2019). The concept has origins in cultural geographer Carl Sauer's (1925) work on the morphology of landscape, which explored how human activities leave a visible record on the landscape, framing landscapes as cultural creations as well as biophysical spaces.

Prominent in Mauro Agnoletti's (2014) and Agnoletti and Rotherham's (2015) work on biocultural diversity in European rural landscapes, the cultural landscape lens highlights how many landscapes that are considered pristine and natural have actually been modified by people. Biodiverse traditional agricultural landscapes were often highlighted as an example of this in the literature on biocultural diversity (Boillat et al., 2013). In this context, cultural landscapes can be defined as areas “where biodiversity [has been] modulated over centuries by traditional agricultural practices” (Vierikko et al., 2020, p. 2). This was linked to the Japanese concept of *satoyama*, which values the unity of nature and culture in rural landscapes and encompasses feelings and emotions (Kieninger et al., 2009). In this context, biocultural diversity is thought to represent broader interactions between people and nature and society and the environment (Dobrovodská et al., 2019), often in human‐modified rural contexts (Agnoletti et al., 2015).

Agnoletti (2014, p. 73) suggested that a biocultural diversity perspective offers a more innovative approach to conservation, which can “shift the focus away from a state of ‘natural’ (or [from] trying to find it in unnatural environments) to examples of positive integration between society and the environment that are occurring in the rural landscape.” This was supported by Cocks and Wiersum (2014), who argue for extending conservation to hybrid nature–culture systems, such as traditional agricultural landscapes, to recognize and value biocultural diversity.

Landscape heritage, including knowledge about the meaning and usefulness of biocultural diversity, is often lost with changes in land use and abandonment of wild plants (Pensado‐Leglise et al., 2022). However, when multidimensional and self‐sustaining, food systems can become “reservoir[s] of biocultural diversity” through localized food harvesting and sharing practices, agrobiodiversity, and alternative production systems, such as meliponiculture, the practice of breeding stingless bees (Aldasoro et al., 2023; Bonicatto et al., 2015; de Pasquale & Spinelli, 2022; Marrero et al., 2023, p. 04). Villodre et al. (2023) argued that the future success of cultural landscape conservation depends on the capacity of public policies to understand, support, and use ILK and beneficial agricultural practices for the maintenance of biocultural diversity. The biocultural diversity framework also offers opportunities for highlighting ethics and social justice in cultural landscape conservation (Cocks et al., 2018).

### Biocultural identity

Identity was positioned as a significant element of biocultural diversity. Cavaliere and Branstrator (2024) present the critical biocultural identity framework, which extends biocultural approaches to conservation by positioning identity as essential to bioregional conservation. Their framework links emotions, identities, and a sense of place and acknowledges how interactions with nature can be influenced by sensory experiences, nonverbal expressions, and emotions, and in turn form emotional attachments. Connection to place is identified as particularly important to forming biocultural identities (Cavaliere & Branstrator, 2024). This added weight to calls for place‐based solutions in biocultural approaches to conservation, which enable biocultural diversity holders to tailor external support to their specific social–ecological contexts (Bridgewater & Rotherham, 2019; Gavin et al., 2018).

The importance of identity in conservation was reflected throughout the papers included in my review. Dyrset et al. (2022) explained that ecological knowledge is intimately linked to people's social identity, their experiences with the natural environment, and their historical rights. Exploring the challenges that come with conserving marine biocultural diversity in Norwegian marine salmon fisheries, Dyrset et al. (2022) found that local fishers identify strongly with their fishing practices and associated knowledge, which they pass down to future generations to continue their sustainable fishing practices and protect their cultural traditions.

Reyes‐Garcia et al. (2023) highlighted the role that culturally important species play not only as indicators of biocultural diversity but also in supporting cultural identity. Identity was considered so important because recovering historical collective memory can provide a way to restore biocultural diversity by reviving traditions, sense of place, identity, and rootedness (Romero Puentes & Rodriguez Susa, 2022). Liu et al. (2023) identified emotions and behaviors as directly affecting the formation of biocultural diversity by forming strong cultural connections to elements of the natural environment.

### Urban people–nature interactions

Biocultural diversity can also include people's relationships with nature in urban areas, a perspective that emerged largely from Michelle Cocks’ work in South Africa. Noting limited critical reflection at the time on biocultural diversity, Cocks and Dold (2006) studied wild plant use in urban areas and found that urbanization does not necessarily imply a loss of traditional values. Based on this, they argued for the recognition of biocultural diversity in urban and peri‐urban contexts, which extends applications of biocultural diversity beyond its initial links with people who live in traditional ways (Cocks, 2006; Cocks & Dold, 2006).

Arguing that conventional definitions of *biocultural diversity* are too narrow, Cocks and Wiersum (2014, p. 733) proposed a complete reappraisal of the concept as “variability among social groups with respect to their value systems, cultural practices, and knowledge systems related to different manifestations of biodiversity.” They contended that biocultural diversity is characterized through cultural ecosystem services and human values and practices that operate in either traditional and rural or modernized and urban societies, adding a caveat that elements of biocultural diversity may not always be conservation oriented (Cocks & Wiersum, 2014).

Urban biocultural diversity is still a developing area of research. Exploring the legacies of charcoal production in Rio de Janeiro, Solórzano et al. (2021) highlighted that the biocultural diversity framework can offer practical solutions for urban forest management through its links with novel ecosystems. Moreover, associated with the European Union‐funded project GREEN SURGE, Elands et al. (2019, p. 33) proposed a distinct new framework for understanding the governance of biocultural diversity in cities, termed “urban biocultural diversity (BCD).” They described the urban BCD framework as providing a move away from a “crisis narrative” about urban areas devoid of connection to the natural world, toward a “dynamic narrative” that highlights coevolutionary interactions between people and nature in cities (Elands et al., 2019, p. 30). Core to this is the understanding that “biodiversity and cultural diversity are inherently connected” (Elands et al., 2019, p. 31). Based on this, the framework does not distinguish between the two but proposes understanding biocultural diversity through lived BCD, materialized BCD, and stewardship BCD:.

“At the heart of the framework is lived BCD, which expresses the day‐to‐day practices and experiences of people interacting with green places, involving, for example, use, emotions, and feelings of belonging. Materialized BCD refers to the tangible manifestation of these interactions, both physically (parks, communal gardens, etc.) and conceptually (contents of management plans, ecosystem services, etc.). Stewardship BCD, finally, includes all forms of engagement in which people take responsibility for the design and management of green areas.” (Pauleit et al., 2019, p. 10)

This is a particularly unique framework because most other definitions distinguished biocultural diversity through the separate elements of biological and cultural diversity. Stålhammar and Brink (2021, p. 602) advocated for the adoption of urban BCD as a distinct concept from “traditional BCD,” designed specifically to capture broader human–nature relationships in urban environments. For example, Vierikko et al. (2020) applied the urban BCD framework to research on the interactions between motivations, experiences, and the environment in public parks. However, Stålhammar and Brink (2021) also expressed concerns that urban BCD simply perpetuates ecosystem services thinking, despite intentions behind the concept to provide an alternative to this (Buizer et al., 2016).

Nonetheless, biocultural diversity is seen as a relevant lens for studying people's interactions with nature in urban spaces. Exploring how biocultural diversity manifests in urban ecosystems, Albuquerque et al. (2023) highlighted urban parks as novel cultural landscapes, botanical gardens and university green spaces, and urban markets and horticulture. They present urban markets as spaces of resistance against agricultural and food homogenization, where urban food sovereignty can be promoted and regional biocultural memory revived (Albuquerque et al., 2023).

### Focus on conserving tradition

Criticisms of biocultural diversity conservation included the concept's focus on conserving traditional ways of living and the assumptions underpinning this; the risk of overgeneralizing biocultural diversity and losing its advocacy potential; discomfort around engaging with biocultural conflicts; and an overall lack of attention to power dynamics in biocultural discourses.

Biocultural diversity research has received criticism for focusing too much on traditional ways of living. Based on work in the Brazilian Pantanal, Arts et al. (2018) argued that biocultural diversity's associations with tradition are problematic because there is a risk of overlooking how cultures evolve and ignoring how many Indigenous Peoples and local communities participate in the globalizing world. Similarly, Albuquerque et al. (2023, p. 3) highlighted how the survival of ILK in urban ecosystems often receives little attention because “knowledge based on traditions is sometimes erroneously perceived by community outsiders and non‐ethnobiologists as ‘static’ or even absent in urban environments.” This reflects wider critical discussion about use of the term *traditional*, which has been framed as colonial due to its overwhelming use to describe non‐Western communities, often overlooking how the cultures in question have changed over time (Mallon [2010] discussing Wendt [1976]).

From a conservation perspective, focusing on tradition in biocultural diversity could be problematic because there is a risk that it may not extend to groups and cultures whose ways of living divert from external expectations of what tradition looks like. For example, working in Guam and Puerto Rico, Marrero et al. (2023) reflected on how farmers and fisherfolk often occupy intermediate, ambiguous positions between their traditional subsistence practices and their engagements with larger labor markets. By overlooking such dynamics, biocultural diversity risks reinforcing “narrow notions of Indigenous identity” (Chiaravalloti, 2019, p. 173), perpetuating expectations for Indigenous Peoples and local communities to behave in certain ways and play roles as particular environmental subjects in order to access conservation protections. For example, in their work on fixing nonmarket subjects, Li (2014) highlighted how initiatives, such as REDD+, entrench expectations that forest communities should not participate in modern economic markets (e.g., through agricultural activity). This links to assumptions within conservation schemes that Indigenous Peoples and local communities are predisposed to living in traditional ways with environmental conservation as “their own authentic goal” (Li, 2014, p. 46). Although participation is framed as a choice, there may not always be another option for people living under conservation schemes, such as REDD+, other than to meet these expectations in order to access the scheme's benefits and protections (Li, 2014). This also reflects concerns by Hill et al. (2011) that biocultural diversity research risks reinforcing networks of unequal power relations, which facilitates the control of diverse Indigenous and local interests and futures for the purposes of others. This is key considering the amount of international funding currently being leveraged toward Indigenous‐led conservation, which risks placing a disproportionate burden for conservation on Indigenous Peoples and local communities.

Although there were examples of biocultural diversity theory shifting away from a focus on tradition such as the development of the urban BCD framework, urban BCD was designed specifically for thinking about governance in cities (Elands et al., 2019, 2015) and is not necessarily suitable for understanding biocultural diversity in a conservation context. For example, testing the application of urban BCD in a Brazilian favela, Stålhammar and Brink (2021) questioned whether the *bio‐* in urban BCD still represents biodiversity or simply reflects nature more generally. This indicates a need for further conceptualization of biocultural diversity for conservation applications, where biodiversity remains highly relevant. In their principles for biocultural conservation, Gavin et al. (2015, p. 142) noted that planning to conserve culture “risks seeking to fix it in place and time.” Cultural identities must be recognized as flexible and fluid, honoring how people's identities may involve moving back and forth between what is considered traditional and modern without questioning the validity of these identities or “reinforcing the already oppressive restrictions placed on local communities that are close to or part of conservation initiatives” (Chiaravalloti, 2019, p. 173). Moreover, Gavin et al. (2015, p. 142) proposed that biocultural approaches to conservation need to allow space for cultural systems to adapt and change with time, including an expectation that “novel and hybrid institutions for the management of diversity” will form.

### Overgeneralization and loss of advocacy function

There was some concern that by reframing biocultural diversity beyond its originally intended applications, the academic community may be “seen to imperialise conceptual territory” (Stålhammar & Brink, 2021, p. 617). Such concerns are not unreasonable because there are documented cases where the conventional biocultural diversity framework has provided an effective protective mechanism for Indigenous Peoples and local communities and their distinct knowledges (Nemogá et al., 2022). Although advocates for reappraising biocultural diversity stressed the need for expanding the concept beyond previously narrow interpretations (e.g., Cocks & Wiersum, 2014), there is a risk that it may become too diluted, potentially losing some of its power as an advocacy tool. This raises ethical questions about appropriating biocultural diversity because biocultural diversity thinking is rooted in many diverse Indigenous epistemologies (Lukawiecki et al., 2022). However, as Davidson‐Hunt et al. (2012) noted, it is a concept developed by scholars to understand nature–culture relations. Davidson‐Hunt et al. (2012) argued that the central question scholars should be asking is how biocultural diversity can guide conservationists to effectively respond to and work with Indigenous Peoples and local communities’ proposals and initiatives within the wider conservation context.

### Neglect of biocultural conflicts

Biocultural relations are not always harmonious. Torrents‐Ticó et al. (2023, p. 435) discussed “biocultural conflicts, where certain cultural practices can lead to biodiversity loss and, in turn, threaten the continuance of such practices.” Although most of the literature on biocultural diversity conservation focused on the synergies between cultural and biological diversities, Torrents‐Ticó et al. (2023) highlighted a need to acknowledge and engage with biocultural conflicts and situations where these synergies may negatively affect people and nature. For example, Torrents‐Ticó et al. (2023) explored concerns among the Kenyan Daasanach community that their use of skins from threatened carnivores in the Dimi ceremony (an important rite of passage for Daasanach agropastoralists) was becoming unsustainable following a reduction in carnivore numbers. They highlighted how younger community members are exploring alternatives, such as storing and reusing existing skins and transitioning to synthetic replicas (Torrents‐Ticó et al., 2023).

This raises interesting questions about where biocultural diversity sits in relation to preserving elements of culture that may cause harm to the environment or be considered harmful in their own right. As biocultural approaches to conservation become more popular in international conservation circles, it is likely that external conservation actors will come across biocultural conflicts and will need to find ways to navigate them. In these situations, it will be important to examine who is deciding which aspects of people's culture are valuable for conservation and whose agendas these decisions serve.

### Lack of attention to power

Reflecting on the wider biocultural paradigm, Merçon et al. (2019) highlighted that biocultural discourses have so far involved limited recognition of power relations. This is problematic, as a lack of attention to power may generate only “partial and naïve understandings of human‐nature interactions” (Merçon et al., 2019, p. 8). On this basis, Merçon et al. (2019, p. 8) propose that

Understanding biocultural systems from a power relations perspective is needed to account for different types of inequities that accentuate cultural groups’ vulnerability in the face of hegemonic cultural, political and economic forces. With such understanding, the biocultural paradigm can be used to counter globalization's homogenizing drivers and the loss of cultural practices, languages, knowledge, values and governance systems. This emancipatory nature of the biocultural paradigm places social and environmental justice at the core of global sustainability.

For example, in Thailand, although many people from Karen communities living in Thung Yai identify as Indigenous, the Thai state's reluctance to acknowledge this, alongside negative stereotyping, has resulted in repeated eviction attempts against the Karen Peoples since Thung Yai's nomination as a World Heritage Site in the 1980s (Buergin, 2015). In addition, the strong association between Indigenous Peoples and biocultural diversity may risk marginalizing rural local communities that have many of the same traits or relationships with nature but are not technically classed as Indigenous. If biocultural diversity is to provide a successful alternative for conservation, then paying attention to power dynamics will be critical to avoid reinforcing preconceptions about how communities should look or act, or going too far the other way and no longer functioning as a protective mechanism.

## DISCUSSION AND RECOMMENDATIONS FOR CONSERVATION ACTORS

**FIGURE 2:**
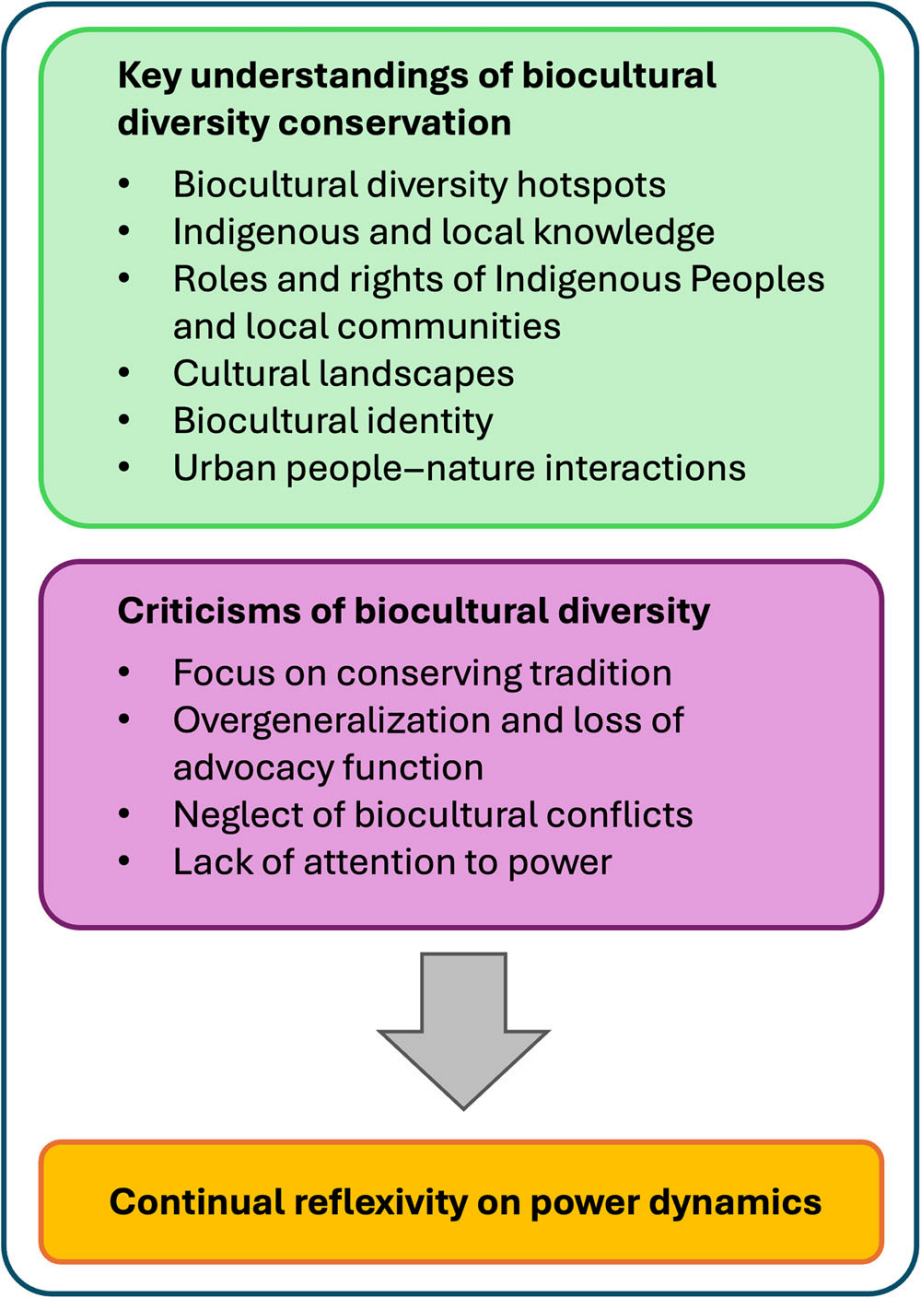
Summary of the key understandings of biocultural diversity conservation, criticisms of how the term *biocultural diversity* is used, and the recommendation for how to move toward a more socially just approach.

Establishing an agenda for conserving biocultural diversity could shift the focus of conservation finance toward initiatives that genuinely protect the interrelations between people and the environment, rather than continuing to reinforce the human–nature dichotomy. However, to harness the potential for external actors to support holders of biocultural diversity as an alternative to mainstream conservation, existing critiques of biocultural diversity must be addressed (Figure 2).

Research on biocultural diversity has often paid too little attention to its political dimensions, despite evidence that the biocultural diversity discourse can provide “an essential political tool to demand land rights protection,” particularly in global South contexts (Merçon et al., 2019, p. 3). Adams and Hutton (2007, pp. 151–152) state that “the way nature is understood has profound political significance” and “the relationship between people and nature…is highly political.” As a conceptual representation of the relationships between people and nature, biocultural diversity is also inherently political. External actors who seek to support biocultural diversity conservation must therefore maintain an awareness of the relevant sociopolitical contexts and associated power dynamics.

Although I focused on biocultural diversity, additional concepts that emerged from my analyses included biocultural heritage (Bridgewater & Rotherham, 2019; Davidson‐Hunt et al., 2012; Frascaroli, 2016), biocultural memory (Fernandez‐Llamazares & Lepofsky, 2019; Gutiérrez‐Santillán et al., 2019), and biocultural ethics (Rozzi et al., 2020). These concepts align with the most common applications of the word *biocultural* in Lukawiecki et al.’s (2022) review of literature on biocultural theory, which also highlighted the concepts of biocultural landscape and biocultural rights. Based on the emergence of various biocultural concepts or “biocultural lenses” (Hanspach et al., 2020, p. 4), Merçon et al. (2019) suggested that a biocultural paradigm is emerging that has distinct ontological, epistemological, and ethicopolitical dimensions. Although it is beyond the scope of my review to delve deeper into the meanings of these lenses, it is important to acknowledge this wider biocultural discourse or otherwise risk losing important contextual understanding about biocultural diversity conservation.

As an emerging approach to conservation, biocultural diversity would benefit from a political ecology perspective that explores how “power and meanings conferred on the landscape play out in the realm of conservation” (Jones, 2006, p. 483). Understandings of biocultural diversity are needed that move away from limiting expectations of tradition (Arts et al., 2018), without taking away from the concept's function as an advocacy tool for some Indigenous Peoples (Nemogá et al., 2022). In practice, biocultural conservation must avoid embedding expectations for Indigenous Peoples and local communities to perform particular homogenized behaviors or identities in order to be deemed valuable by global actors (Li, 2014) or forcing institutional visibility on communities and “instrumentaliz[ing] Indigenous ‘traditions’ in service to [conservation]” (Rubis & Theriault, 2020, p. 978). In response to these needs, a political ecology of biocultural diversity could generate huge potential for a more socially just approach to conservation.

I designed the prompts below in response to the criticisms I identified (Table 3). They are intended to encourage external actors who seek to influence or support biocultural conservation to be more attentive to power dynamics as they engage with on‐the‐ground holders of biocultural diversity. Ongoing interrelations between Indigenous and local actors and their environments are key to biocultural diversity conservation, and external actors can at best support or undermine these dynamics. Continually referring to these questions can help prevent external actors from reproducing the same injustices as previous community conservation interventions (e.g., Dressler et al., 2010; Jones, 2006; Neumann, 1992).

**TABLE 3:** Guiding prompts for external conservation actors to encourage continual reflexivity on power dynamics when seeking to support biocultural diversity conservation.

Addressing critiques of biocultural diversity	Prompts for external conservation actors
Shifting expectations about tradition	What preconceptions do external actors hold about culture and identity in this context? Does the policy or project create space to recognize culture and identity as complex and dynamic? Does the external actor's understanding of biocultural diversity include blanket assumptions about Indigenous Peoples and local communities as noble environmental stewards, or about local resource use as destructive to natural processes? What problems might this create or reinforce? Are external actors expecting people to live or behave according to tradition? How can these expectations be adjusted?
Retaining advocacy potential	Does this initiative respond to proposals or calls from on‐the‐ground holders of biocultural diversity? If not, how can external actors reorient the goals of the initiative to reflect this? Is this initiative led by Indigenous Peoples or local communities? DoIndigenous Peoples and local communities have decision‐making power in the context within which they are working?
Acknowledging biocultural conflicts	How will biocultural relationships that might be causing social or environmental harm be navigated, if encountered?
Attention to power	What are the power dynamics or tensions at play in this context? What are the risks of reproducing or feeding into these dynamics? How can these risks be addressed or accounted for? Who decides which types of biocultural diversity are considered valuable within this initiative? Who controls where funding is being directed? Who will benefit from this initiative? Who will be excluded or otherwise negatively affected? What can external actors do to mitigate the latter? Is there a risk of undermining the work of on‐the‐ground holders of biocultural diversity? What can external actors do to mitigate this?

These prompts (Table 3) can help external actors maintain respect for local actors who materially create and conserve biocultural diversity and mitigate expectations for biocultural diversity conservation to look the same way in different settings. For instance, biocultural diversity has been mobilized by Indigenous civil society organizations around the world to secure funding from the GEF‐7 Inclusive Conservation Initiative (ICI, 2024). In Kenya, for example, this includes supporting the development of biocultural community protocols to improve land tenure security and resource access for pastoralist communities. Although the project is led by an Indigenous civil society organization, employs ILK, and seeks recognition for a cultural landscape, it is ultimately funded by a huge multilateral organization. As a result, paying attention to the contextual power dynamics at play is critical to prevent performative or reductive applications of biocultural diversity being imposed by funding and implementation teams. The prompts are intended to guide such external actors in this reflexive practice (Table 3).

Although transformative change is ultimately needed for just conservation (Massarella et al., 2022) and available funding for biocultural conservation initiatives remains limited, there may also be opportunities to integrate biocultural diversity into existing conservation spaces, reorienting conservation priorities to mitigate further enforcement of human–nature divisions. Although devised to guide biocultural diversity conservation initiatives, the prompts are also relevant for mainstream conservation actors who seek to be more inclusive of people's interactions and interrelations with the environment. In such cases, it will be important to consider whose cultures and biocultural relations are valued and whose are dismissed. These prompts are also relevant for actors promoting biocultural approaches to restoration, such as projects in which ILK is used to guide species selection in restoration processes (Sena et al., 2022).

Biocultural diversity theory has similarities with other alternative and radical visions for conservation, such as convivial conservation (Herse, 2022; Pettersson et al., 2022) and decolonial conservation (Kashwan et al., 2021; Townsend & Roth, 2023). For instance, biocultural diversity conservation has a central focus on protecting the rights of Indigenous Peoples and local communities; challenges the human–nature dichotomy by seeing people and nature as inextricably linked; and recognizes a plurality of worldviews and values ILK, helping to address epistemic injustices (Massarella et al., 2022). Just as more attention to the dimensions of social justice is recommended for convivial conservation to be feasible in Global South contexts (Kiwango & Mabele, 2022), the biocultural paradigm was proposed as a useful approach for integrating social justice principles into sustainability (Merçon et al., 2019). There is an imperative for further research that considers how these alternative conservation approaches interact and complement one another.

As biocultural diversity gains attention on a global scale, it will be important to consider how these different terminologies are being politically mobilized toward similar ends, including to gain access to conservation finance. With this in mind, there is a risk that biocultural diversity could become reduced to a buzzword by external actors, for instance, if it is adopted by high‐profile organizations to gain traction and support without engaging with on‐the‐ground holders of biocultural diversity and in so doing losing its transformative potential (Chandhoke [2007] in Massarella et al. [2022]). Practicing continual reflexivity by exploring how power dynamics unfold will therefore be critical for external actors.

My findings serve as a warning for external actors seeking to influence biocultural diversity conservation and are consistent with calls for better attention to power and politics in biocultural approaches to conservation. Extending support to on‐the‐ground holders of biocultural diversity in ways that are inclusive and socially just will require continual, meaningful reflection on power dynamics by both conservation researchers and practitioners. It will also require these reflections to be translated into action, making adjustments in practice to account for risks identified and to mitigate injustices. If conservation actors are willing to engage in this reflexive practice, such as by following the guiding prompts presented here, biocultural diversity could have huge potential for transforming the way conservation is thought about and enacted at global scales. This includes overcoming the human–nature divide by backing conservation methodologies that genuinely value peoples’ interactions and interrelations with the rest of nature, respecting and supporting on‐the‐ground actors to achieve global biodiversity goals while upholding principles of social and environmental justice (Kiwango & Mabele, 2022; Massarella et al., 2022). Such transformative change will be critical for realizing the CBD 2050 Vision of living in harmony with nature.

## Supporting information

Supporting Information
