# Supplementary material for: Understandings and critiques of biocultural diversity conservation and future recommendations for conservation actors
Source: Conserv Biol. 2025 Aug 24;40(1):e70131. doi: 10.1111/cobi.70131 (PMC12856825; doi:10.1111/cobi.70131)
Supplement: Supplementary file 1 — Supporting Information [file COBI-40-e70131-s001.docx]

# Supporting Information

Appendix S1. List of studies that defined or provided a framework for biocultural diversity in relation to environmental conservation*.*

Agnoletti, M. & Rotherham, I. D. (2015). Landscape and biocultural diversity. *Biodiversity and Conservation, 24,* 3155–3165.

Agnoletti, M. (2014). Rural landscape, nature conservation and culture: Some notes on research trends and management approaches from a (southern) European perspective. *Landscape and Urban Planning*, *126*, 66-73.

Agnoletti, M., Tredici, M., & Santoro, A. (2015). Biocultural diversity and landscape patterns in three historical rural areas of Morocco, Cuba and Italy. *Biodiversity and Conservation*, *24*, 3387-3404.

Albuquerque, U., Ladio, A., Almada, E., Vandebroek, I., Silva, M. T. P., & Fonseca-Kruel, V. S. (2023). Exploring biocultural diversity in urban ecosystems: an ethnobiological perspective. *Ethnobiology and Conservation*, *12.*

Aldasoro Maya, E. M., Rodríguez Robles, U., Martínez Gutiérrez, M. L., Chan Mutul, G. A., Avilez López, T., Morales, H., Ferguson, B.G., & Mérida Rivas, J. A. (2023). Stingless bee keeping: Biocultural conservation and agroecological education. *Frontiers in Sustainable Food Systems*, *6*, 1081400.

Apgar, J. M., Ataria, J. M., & Allen, W. J. (2011). Managing beyond designations: supporting endogenous processes for nurturing biocultural development. *International Journal of Heritage Studies*, *17*(6), 555-570.

Argumedo, A., Song, Y., Khoury, C. K., Hunter, D., Dempewolf, H., Guarino, L., & de Haan, S. (2021). Biocultural diversity for food system transformation under global environmental change. *Frontiers in Sustainable Food Systems*, *5*, 685299.

Arts, K., Rabelo, M. T. O., De Figueiredo, D. M., Maffey, G., Ioris, A. A. R., & Girard, P. (2018). Online and offline representations of biocultural diversity: A political ecology perspective on nature-based tourism and indigenous communities in the Brazilian Pantanal. *Sustainability*, *10*(10), 3643.

Axelsson, E. P., & Franco, F. M. (2023). Popular Cultural Keystone Species are also understudied—the case of the camphor tree (Dryobalanops aromatica). *Trees, Forests and People*, *13*, 100416.

Boillat, S., Mathez-Stiefel, S. L., & Rist, S. (2013). Linking local knowledge, conservation practices and ecosystem diversity: comparing two communities in the Tunari National Park (Bolivia). *Ethnobiology and Conservation*, *2.*

Bonicatto, M. M., Marasas, M. E., Sarandon, S., & Pochettino, M. L. (2015). Seed conservation by family farmers in the rural–urban Fringe Area of La Plata Region, Argentina: the dynamics of an ancient practice. *Agroecology and Sustainable Food Systems*, *39*(6), 625-646.

Bridgewater, P., & Rotherham, I. D. (2019). A critical perspective on the concept of biocultural diversity and its emerging role in nature and heritage conservation. *People and Nature*, *1*(3), 291-304.

Briggs, L., Stedman, R., & Krasny, M. (2019). Place attachment and social–ecological system sustainability examined through the voices of indigenous Guatemalan women. *Sustainability Science*, *14*, 655-667.

Brosius, J. P. & Hitchner, S. L. (2010). Cultural diversity and conservation. *International Social Science Journal*, *61*, 141–168.

Buergin, R. (2015). Contested rights of local communities and indigenous Peoples in conflicts over biocultural diversity: the case of Karen communities in Thung Yai, a world heritage site in Thailand. *Modern Asian Studies*, *49*(6), 2022-2062.

Buizer, M., Elands, B., & Vierikko, K. (2016). Governing cities reflexively—The biocultural diversity concept as an alternative to ecosystem services. *Environmental Science & Policy*, *62*, 7-13.

Cámara-Leret, R., & Bascompte, J. (2021). Language extinction triggers the loss of unique medicinal knowledge. *Proceedings of the National Academy of Sciences*, *118*(24), e2103683118.

Cavaliere, C. T., & Branstrator, J. R. (2024). A Critical Biocultural Identity Framework. *Society & Natural Resources*, *37*(2), 213-233.

Chengere, S. A., Steger, C., Gebrehiwot, K., Nemomissa, S., & Dullo, B. W. (2022). Modeling cultural keystone species for the conservation of biocultural diversity in the Afroalpine. *Environments*, *9*(12), 156.

Cocks, M. (2006). Biocultural diversity: moving beyond the realm of ‘indigenous’ and ‘local’ people. *Human Ecology*, *34*(2), 185-200.

Cocks, M. L. & Dold, A. P. (2006). Cultural significance of biodiversity: The role of medicinal plants in Urban African cultural practices in the Eastern Cape, South Africa. *Journal of Ethnobiology*, *26,* 60–81.

Cocks, M. L. & Wiersum, F. (2014). Reappraising the concept of biocultural diversity: A perspective from South Africa, *Human Ecology*, *42*, 727–737.

Cocks, M., Alexander, J., Mogano, L. & Vetter, S. (2016). Ways of belonging: Meanings of “nature” among Xhosa-speaking township residents in South Africa*, Journal of Ethnobiology*, *36*, 820–841.

Cocks, M., Vetter, S. & Wiersum, K. F. (2018). From universal to local: Perspectives on cultural landscape heritage in South Africa. *International Journal of Heritage Studies*, *24*, 35–52.

Davidson-Hunt, I. J., Turner, K. L., Mead, A. T. P., Cabrera-Lopez, J., Bolton, R., Idrobo, C. J., Miretski, I., Morrison, A., & Robson, J. P. (2012). Biocultural design: a new conceptual framework for sustainable development in rural indigenous and local communities. *Sapiens. Surveys and Perspectives Integrating Environment and Society*, *5*(2), 33-45.

de Pasquale, G., & Spinelli, E. (2022). The alpine rural landscape as a cultural reserve: the case study of Teglio in Valtellina. *Biodiversity and Conservation*, *31*(10), 2397-2420.

Díaz, S., Demissew, S., Carabias, J., Joly, C., Lonsdale, M., Ash, N., Larigauderie, A., Adhikari, J.R., Arico, S., B ́aldi, A., Bartuska, A., Baste, I.A., Bilgin, A., Brondizio, E., Chan, K.M.A., Figueroa, V.E., Duraiappah, A., Fischer, M., Hill, R., … & Zlatanova, D. (2015). The IPBES Conceptual Framework—connecting nature and people. *Current Opinion in Environmental Sustainability*, *14*, 1-16.

Dobrovodská, M., Kanka, R., David, S., Kollár, J., Špulerová, J., Štefunková, D., Mojses, M., Petrovič, F., Krištín, A., Stašiov, S., Halada, Ľ., & Gajdoš, P. (2019). Assessment of the biocultural value of traditional agricultural landscape on a plot-by-plot level: case studies from Slovakia. *Biodiversity and Conservation*, *28*, 2615-2645.

Doda, Z. (2019). The conservation of African yellowwood tree (Afrocarpus falcatus) in Sidama sacred sites, Ethiopia. *Cogent Social Sciences,* *5*, 1–17.

Dyrset, G., Margaryan, L., & Stensland, S. (2022). Local knowledge, social identity and conflicts around traditional marine salmon fisheries–A case from Mid‐Norway. *Fisheries Management and Ecology*, *29*(2), 131-142.

Elands, B. H. M., Vierikko, K., Andersson, E., Fischer, L. K., Goncalves, P., Haase, D., Kowarik, I., Luz, A.C., Niemelä, J., Santos-Reis, M., & Wiersum, K. F. (2019). Biocultural diversity: A novel concept to assess human-nature interrelations, nature conservation and stewardship in cities. *Urban Forestry & Urban Greening*, *40*, 29-34.

Elands, B. H. M., Wiersum, K. F., Buijs, A. E., & Vierikko, K. (2015). Policy interpretations and manifestation of biocultural diversity in urbanized Europe: conservation of lived biodiversity. *Biodiversity and Conservation*, *24*, 3347-3366.

Eriksson, O. (2018) What is biological cultural heritage and why should we care about it? An example from Swedish rural landscapes and forests. *Nature Conservation*, *28*, 1–32.

Fernandez-Llamazares, A. & Lepofsky, D. (2019). Ethnobiology through song. *Journal of Ethnobiology, 39,* 337–353.

Franco, F. M. (2022). Ecocultural or biocultural? Towards appropriate terminologies in biocultural diversity. *Biology*, *11*(2), 207.

Franco-Moraes, J., Braga, L. V., & Clement, C. R. (2023). The Zoʻé perspective on what scientists call “forest management” and its implications for floristic diversity and biocultural conservation. *Ecology and Society*, *28*(1).

Frascaroli, F. (2016). Shepherds, rituals, and the sacred: A biocultural view of the non-modern ontologies of folk shrines and devotions in Central Italy. *Worldviews: Global Religions, Culture, and Ecology*, *20*(3), 272-285.

Gavin, M. C., McCarter, J., Berkes, F., Mead, A. T. P., Sterling, E. J., Tang, R., & Turner, N. J. (2018). Effective biodiversity conservation requires dynamic, pluralistic, partnership-based approaches. *Sustainability*, *10*(6), 1846.

Gavin, M. C., McCarter, J., Mead, A., Berkes, F., Stepp, J. R., Peterson, D., & Tang, R. (2015). Defining biocultural approaches to conservation. *Trends in ecology & evolution*, *30*(3), 140-145.

Gutiérrez-Santillán, T. V., Moreno-Fuentes, Á., Sánchez-González, A., & Sanchez-Rojas, G. (2019). Knowledge and use of biocultural diversity by Nahua in the Huasteca region of Hidalgo, Mexico. *Ethnobiology and Conservation*, *8*.

Harmon, D. (2007) A Bridge over the Chasm: Finding Ways to Achieve Integrated Natural and Cultural Heritage Conservation. *International Journal of Heritage Studies*, *13*(4-5), 380-392.

Hill, R., Cullen-Unsworth, L. C., Talbot, L. D., & McIntyre-Tamwoy, S. (2011). Empowering Indigenous Peoples’ biocultural diversity through World Heritage cultural landscapes: a case study from the Australian humid tropical forests. *International Journal of Heritage Studies*, *17*(6), 571-591.

Hill, R., Nates-Parra, G., Quezada-Euán, J. J. G., Buchori, D., LeBuhn, G., Maués, M. M., Pert, P.L., Kwapong, P.K., Saeed, S., Breslow, S.J., Carneiro da Cunha, M., Dicks, L.V., Galetto, L., Gikungu, M., Howlett, B.G., Imperatriz-Fonseca, V.L., O’B. Lyver, P., Martín-López, B., Oteros-Roza, E., … & Roué, M. (2019). Biocultural approaches to pollinator conservation. *Nature Sustainability*, *2*(3), 214-222.

Hong, S. K. (2013). Biocultural diversity conservation for island and islanders: Necessity, goal and activity. *Journal of Marine and Island Cultures,* *2,* 102–106.

Hong, S. K., Won, Y. T., Lee, G. A., Han, E. S., Cho, M. R., Park, H. Y., Kim, J. E., & Chisholm Hatfield, S. (2018). Interdisciplinary convergence research design on island biocultural diversity-Case study in Wando-gun (County) Island region, South Korea. *Journal of Marine and Island Cultures, 7*(1).

Hughes, H. & Vadrot, A.B.M. (2019). Weighting the world: IPBES and the struggle over biocultural diversity. *Global Environmental Politics*, *19*, 14–37.

Kieninger, P., Holzner, W., & Kriechbaum, M. (2009). Emotions and the Fun-Factor in Nature Conservation–a Lesson from Japan. *Bodenkultur*, *60,* 15–21.

Kulak, V., Longboat, S., Brunet, N. D., Shukla, M., & Saxena, P. (2022). In vitro technology in plant conservation: Relevance to biocultural diversity. *Plants*, *11*(4), 503.

Laird, S., Awung, G., Lysinge, R. & Ndive, L. (2011). The interweave of people and place: Biocultural diversity in migrant and indigenous livelihoods around Mount Cameroon. *International Forestry Review,* *13*, 275–293.

Latorre, E. C., Canavero, A., & Pochettino, M. L. (2018). Comparison of medicinal plant knowledge between rural and urban people living in the Biosphere Reserve “Bioma Pampa-Quebradas del Norte”, Uruguay: an opportunity for biocultural conservation. *Ethnobiology and Conservation*, *7*(4).

Linares-Rosas, M. I., Gómez, B., Aldasoro-Maya, E. M., & Casas, A. (2021). Nahua biocultural richness: an ethnoherpetological perspective. *Journal of Ethnobiology and Ethnomedicine*, *17*(1), 33.

Liu, S., Zhang, Z., Jiao, Y., Shi, W., Liu, N., & Wang, L. (2023). Spatial coupling mechanism of biocultural diversity: case of Beichuan-River-Source National Nature Reserve, Qinghai, China. *International Journal of Sustainable Development & World Ecology*, *30*(4), 458-484.

Loh, J. & Harmon, D. (2005). A global index of biocultural diversity. *Ecological Indicators,* *5*, 231–241.

Lukawiecki, J., Wall, J., Young, R., Gonet, J., Azhdari, G. & Moola, F. (2022). Operationalizing the biocultural perspective in conservation practice: A systematic review of the literature. *Environmental Science and Policy, 136,* 369–376.

Maffi, L. (2002). Endangered languages, endangered knowledge. *International Social Science Journal,* *54*, 385–393.

Maffi, L. (2005). Linguistic, Cultural, and Biological Diversity. *Annual Review of Anthropology,* *29*, 599–617.

Marrero, A., Nicoson, C., & Mattei, J. (2023). Food laborers as stewards of island biocultural diversity: reclaiming local knowledge, food sovereignty, and decolonization. *Frontiers in Sustainable Food Systems*, *7*, e1093341.

Merçon, J., Vetter, S., Tengö, M., Cocks, M., Balvanera, P., Rosell, J. A., & Ayala-Orozco, B. (2019). From local landscapes to international policy: contributions of the biocultural paradigm to global sustainability. *Global Sustainability*, *2*, e7.

Min, Q., Yang, Xiao., & Ding, L. (2022). The concept, connotation and significance of cultural keystone species in agricultural heritage systems. *Journal of resources and ecology*, *13*(1), 51-60.

Monroy-Sais, S., García-Frapolli, E., Casas, A., Mora, F., Skutsch, M., & Gerritsen, P. R. (2022). Relational values and management of plant resources in two communities in a highly biodiverse area in western Mexico. *Agriculture and Human Values*, *39*(4), 1231-1244.

Mooij, M. L., Dessartre Mendonça, S., & Arts, K. (2018). Conserving Biocultural Diversity through Community–Government interaction: a practice-based approach in a Brazilian extractive reserve. *Sustainability*, *11*(1), 32.

Nemogá, G. R. (2016). Biocultural diversity: Innovating in research for conservation. *Acta Biologica Colombiana, 21*, S311–S319.

Nemogá, G. R., Appasamy, A., & Romanow, C. A. (2022). Protecting indigenous and local knowledge through a biocultural diversity framework. *The Journal of Environment & Development*, *31*(3), 223-252.

Oloriz, C., & Parlee, B. (2020). Towards biocultural conservation: Local and indigenous knowledge, cultural values and governance of the White Sturgeon (Canada). *Sustainability*, *12*(18), e7320.

Pauleit, S., Ambrose-Oji, B., Andersson, E., Anton, B., Buijs, A., Haase, D., Elands, B., Hansen, R., Kowarik, I., Kronenberg, J., Mattijssen, T., Stahl Olafsson, A., Rall, E., van der Jagt, A. P. N., & van den Bosch, C. K. (2019). Advancing urban green infrastructure in Europe: Outcomes and reflections from the GREEN SURGE project. *Urban forestry & urban greening*, *40*, 4-16.

Pensado-Leglise, M. D. R., Luna-Vargas, S., & Bustamante-Ramírez, H. A. (2022). Conservation of biocultural diversity in the Huasteca Potosina region, Mexico. *Diversity*, *14*(10), 841.

Pert, P. L., Hill, R., Maclean, K., Dale, A., Rist, P., Schmider, J., Talbot, L., & Tawake, L. (2015). Mapping cultural ecosystem services with rainforest aboriginal peoples: Integrating biocultural diversity, governance and social variation. *Ecosystem Services*, *13*, 41-56.

Pettersson, H. L., Quinn, C. H., Holmes, G., & Sait, S. M. (2022). “They Belong Here”: Understanding the Conditions of Human-wolf Coexistence in North-Western Spain. *Conservation and Society*, *20*(2), 113-123.

Plieninger, T., Kohsaka, R., Bieling, C., Hashimoto, S., Kamiyama, C., Kizos, T., Penker, M., Kieninger, P., Shaw, B. J., Sioen, G. B., Yoshida, Y. & Saito, O. (2018). Fostering biocultural diversity in landscapes through place-based food networks: A “solution scan” of European and Japanese models. *Sustainability Science*, *13*, 219–233.

Prakofjewa, J., Sartori, M., Šarka, P., Kalle, R., Pieroni, A., & Sõukand, R. (2023). Boundaries Are Blurred: Wild Food Plant Knowledge Circulation across the Polish-Lithuanian-Belarusian Borderland. *Biology*, *12*(4), 571.

Reyes-García, V., Cámara-Leret, R., Halpern, B. S., O’hara, C., Renard, D., Zafra-Calvo, N., & Díaz, S. (2023). Biocultural vulnerability exposes threats of culturally important species. *Proceedings of the National Academy of Sciences*, *120*(2), e2217303120.

Robertson, D. P. & Hull, R. B. (2003). Biocultural ecology: Exploring the social construction of the Southern Appalachian Ecosystem. *Natural Areas Journal,* *23*, 180–189.

Romero Puentes, R. B., & Rodríguez Susa, M. S. (2022). Old trees, sprouts, and seeds of the cloud forest: the voices of the campesinos. *Ecology and Society*, *27*(4). <https://doi.org/10.5751/ES-13539-270446>.

Rozzi, R. (2012). Biocultural ethics: recovering the vital links between the inhabitants, their habits, and habitats. *Environmental Ethics*, *34*(1), 27-50.

Rozzi, R. (2012). Biocultural ethics: recovering the vital links between the inhabitants, their habits, and habitats. *Environmental Ethics*, *34*(1), 27-50.

Rozzi, R., Álvarez, R., Castro, V., Núñez, D., Ojeda, J., Tauro, A., & Massardo, F. (2023). Biocultural calendars across four ethnolinguistic communities in southwestern South America. *GeoHealth*, *7*(4), e2022GH000623.

Rozzi, R., Arango, X., Massardo, F., Anderson, C., Heidinger, K. & Moses, K. (2008). Field environmental philosophy and biocultural conservation: The Omora Ethnobotanical Park educational program. *Environmental Ethics*, *30,* 325–336.

Rozzi, R., Massardo, F., Anderson, C. B., Heidinger, K. & Silander Jr., J. A. (2006). Ten Principles for Biocultural Conservation at the Southern Tip of the Americas: The Approach of the Omora Ethnobotanical Park. *Ecology and Society 11*(1), 43.

Seele, B. C., Esler, K. J. & Cunningham, A. B. (2019). Biocultural diversity: A mongolian case study. *Ecology and Society,* *24*(4), 27.

Sena, P. H., Gonçalves‐Souza, T., Gonçalves, P. H., Ferreira, P. S., Gusmão, R. A., & Melo, F. P. (2022). Biocultural restoration improves delivery of ecosystem services in social‐ecological landscapes. *Restoration Ecology*, *30*(5), e13599.

Simms, S. R., & Porter-Bolland, L. (2022). Local ecological knowledge of beekeeping with stingless bees (Apidae: Meliponini) in Central Veracruz, Mexico. *Journal of Apicultural Research*, *61*(5), 717-729.

Singh, R. K., Kumar, A., Singh, A. & Singhal, P. (2020). Evidence that cultural food practices of Adi women in Arunachal Pradesh, India, improve social-ecological resilience: Insights for Sustainable Development Goals. *Ecological Processes,* *9,* 29.

Singh, R., Pretty, J. & Pilgrim, S. (2010) Traditional knowledge and biocultural diversity: Learning from tribal communities for sustainable development in northeast India, *Journal of Environmental Planning and Management,* 53: 511–533.

Sirakova, S. M. (2023). Forgotten Stories of Yogurt: Cultivating Multispecies Wisdom. *Journal of Ethnobiology*, *43*(3), 250-261.

Soldal, H., Múrcia, C., Ouhammou, A., Hawkins, J. A., Martin, G. J., Puri, R. K., & Teixidor-Toneu, I. (2023). Plant names encode Tašlḥit knowledge of Morocco’s high atlas landscapes. *Human Ecology*.

Solórzano, A., Brasil-Machado, A., & Ribeiro de Oliveira, R. (2021). Land use and social-ecological legacies of Rio de Janeiro's Atlantic urban forests: from charcoal production to novel ecosystems. *Royal Society Open Science*, *8*(6), e201855.

Spirito, F., Vieli, L., & Montalba, R. (2022). Advancing towards an understanding of the relationship between culture and agrobiodiversity. A case study in Mapuche territory, southern Chile. *NJAS: Impact in Agricultural and Life Sciences*, *94*(1), 1-23.

Stålhammar, S., & Brink, E. (2021). ‘Urban biocultural diversity’as a framework for human–nature interactions: reflections from a Brazilian favela. *Urban Ecosystems*, *24*(3), 601-619.

Torrents-Ticó, M., Fernández-Llamazares, Á., Burgas, D., Nasak, J. G., & Cabeza, M. (2023). Biocultural conflicts: understanding complex interconnections between a traditional ceremony and threatened carnivores in north Kenya. *Oryx*, *57*(4), 435-444.

Turner, N. J., Cuerrier, A., & Joseph, L. (2022). Well grounded: Indigenous Peoples' knowledge, ethnobiology and sustainability. *People and Nature*, *4*(3), 627-651.

Turvey, S. T. & Pettorelli, N. (2014). Spatial congruence in language and species richness but not threat in the world’s top linguistic hotspot. *Proceedings of the Royal Society B: Biological Sciences*, *281*, 20141644.

Tydecks, L., Hernández-Agüero, J. A., Böhning-Gaese, K., Bremerich, V., Jeschke, J. M., Schütt, B., Zarfl, C., & Tockner, K. (2023). Oases in the Sahara Desert–Linking biological and cultural diversity. *Plos One*, *18*(8), e0290304.

Vidal, O., & Brusca, R. C. (2020). Mexico’s biocultural diversity in peril. *Revista de Biología Tropical*, *68*(2), 669-691.

Vierikko, K., Gonçalves, P., Haase, D., Elands, B., Ioja, C., Jaatsi, M., Pieniniemi, M., Lindgren, J., Grilo, F., Santos-Reis, M., Niemelä, J., & Yli-Pelkonen, V. (2020). Biocultural diversity (BCD) in European cities–Interactions between motivations, experiences and environment in public parks. *Urban Forestry & Urban Greening*, *48*, e126501.

Villodre, M., Arnaiz-Schmitz, C., & Schmitz, M. F. (2023). Landscape conservation in the natural-rural interface. A social-ecological approach in Natural Parks of Andalusia (Spain). *Landscape Ecology*, *38*(12), 3517-3535.
